# Expanding the Clinical and Molecular Spectrum of Primary Autosomal Recessive Microcephaly: Novel CDK5RAP2 Gene Variants and Functional Insights on the Intronic Variants

**DOI:** 10.3390/genes16101120

**Published:** 2025-09-23

**Authors:** Burcu Yeter, Yasemin Kendir Demirkol, Esra Usluer, İpek Görüşen Kavak, Sena Gjota Ergin, Nursel H. Elçioğlu

**Affiliations:** 1 Department of Pediatric Genetics, Umraniye Training and Research Hospital, University of Health Sciences, Istanbul 34764, Türkiye; 2 Department of Pediatrics, School of Medical, Marmara University, Istanbul 34899, Türkiye; 3 Intergen Genetics and Rare Disease Diagnosis Center, R&D Department, Ankara 06510, Türkiye; 4 Department of Pediatric Genetics, School of Medical, Marmara University, Istanbul 34899, Türkiye; 5 Department of Pediatric Genetics, School of Medical, Eastern Mediterranean University, Mersin 99628, Türkiye

**Keywords:** autosomal recessive primary microcephaly 3, *CDK5RAP2*, MCPH3, primary microcephaly, rare disease, whole-exome sequencing

## Abstract

**Background/Objectives**: Autosomal recessive primary microcephaly is a rare and genetically heterogeneous disorder characterized by congenital non-syndromic microcephaly, with at least 28 causative genes identified to date. Biallelic variants in the *CDK5RAP2* gene, an ultra-rare cause of autosomal recessive primary microcephaly, lead to Primary Autosomal Recessive Microcephaly 3 (MCPH3). **Methods**: We present seven patients from six families diagnosed with MCPH3 in light of clinical and molecular findings using whole-exome sequencing (WES). Furthermore, we investigated the effects of the identified intronic variants on splicing through RNA analysis. **Results**: Almost all patients had severe microcephaly, mild to moderate intellectual disability, speech delay, and cutaneous pigmentary abnormalities. Four patients presented with postnatal short stature, and two showed weight deficiency. Dysmorphic evaluation revealed that the most prominent features included brachycephaly, hypertelorism, epicanthus, high-arched eyebrows, prominent nasal bridge, and micrognathia. We identified five distinct homozygous CDK5RAP2 variants in our patients, including four novel variants. Segregation analysis verified that the parents were carriers. Two of these variants were intronic (c.3148+5G>C and c.383+4dupA), two were frameshift (c.3168del), and one was a nonsense variant (c.1591C>T). Both intronic variants disrupted splicing, generating a premature stop codon and resulting in a truncated protein. **Conclusions**: This study broadens the mutational landscape of *CDK5RAP2*. We also sought to demonstrate the functional consequences of the *CDK5RAP2* intronic variants on gene function using RNA analysis. The identification of four novel variants underscores the importance of molecular diagnostics in patients with primary microcephaly and provides valuable data for genetic counseling and future functional studies.

## 1. Introduction

Microcephaly is defined as a noticeable reduction in head circumference, measuring more than two standard deviations (SDs) below the average for a person’s sex, age, and ethnicity, with severe cases defined as more than three SDs below [1]. Microcephaly is categorized as primary (congenital) or secondary (postnatal) based on the timing of its onset. Primary microcephaly results from impaired brain growth during prenatal development, whereas secondary microcephaly is characterized by postnatal onset of progressive brain volume loss following an initially normal brain size [2]. Although there is some overlap, these two categories remain largely distinct, each exhibiting characteristic patterns of impaired brain growth [3]. Primary microcephaly (PM), defined as being present at birth, may result from genetic causes or environmental exposures such as infections (e.g., Zika virus), perinatal hypoxia, hypoglycemia, toxins, radiation, or alcohol [4,5]. According to the Human Phenotype Ontology (HPO), approximately 1300 genes and 1800 genetic disorders have been associated with microcephaly. PM is classified into syndromic and non-syndromic forms. The majority of non-syndromic PM is inherited in an autosomal recessive manner and is referred to as microcephaly primary hereditary (MCPH) [5]. The prevalence of MCPH varies significantly, from around 1 in 10,000 in populations with high consanguinity rates to approximately 1 in 250,000 in the general population [6]. Since the identification of MCPH1 as the first gene associated with MCPH in 1998, a total of 28 genes have been implicated in the etiology of MCPH to date, including two exhibiting autosomal dominant inheritance [7]. Despite the involvement of numerous genes, affected individuals are clinically and phenotypically indistinguishable, underscoring the homogeneity of the MCPH phenotype. Among these, the most frequently identified genes are *ASPM*, *WDR62*, and *MCPH1*, respectively, whereas *CDK5RAP2* is among the more rarely detected genes [8,9]. Homozygous or compound heterozygous variants in the *CDK5RAP2* gene cause Primary Autosomal Recessive Microcephaly 3 (MCPH3) (MIM # 604804).

MCPH3 is a very rare neurodevelopmental disorder caused by biallelic variants in the *CDK5RAP2* gene and is characterized by severe microcephaly, speech delay, learning disabilities, sensorineural hearing loss, and cutaneous pigmentary abnormalities [10]. In addition, neuromotor developmental delay, short stature, feeding difficulties, growth retardation, seizures, spasticity, and ataxia have also been reported in patients with MCPH3 [10,11,12,13,14]. To date, approximately 50 patients with MCPH3 have been reported. Most of the patients described in the literature originate from Pakistan and Saudi Arabia.

In this study, we aimed to evaluate the clinical and molecular characteristics of seven MCPH3 patients with four novel *CDK5RAP2* variants, diagnosed through whole-exome sequencing (WES). In addition, we aimed to assess the impact of the intronic *CDK5RAP2* variants at positions +4 and +5 on protein function by analyzing their effect on splicing through RNA analysis.

## 2. Materials and Methods

This study included 7 Turkish MCPH3 patients from 6 different families referred to the Pediatric Genetics Department due to severe microcephaly, intellectual disability, and speech delay. Chromosome and microarray analyses were performed on peripheral blood of all patients. Afterward, all patients underwent WES. Relevant clinical and laboratory findings were retrieved from past medical records. Written informed consent for genetic testing, as well as for the publication of clinical data, patient photographs, and genetic findings, was obtained from the parents of each patient.

### 2.1. DNA Isolation and WES

Genomic DNA was isolated from peripheral blood utilizing tools and a DNA Blood 500 µL kit from Xiamen Zeesan Biotech (Xiamen, China) in accordance with standard procedures. Whole exome capture and sequencing were performed using a Twist Human Core Exome+ Refseq Kit (South San Francisco, CA, USA). The sequencing reaction was performed using reagent kits compatible with the Illumina NextSeq system. Libraries were sequenced using a NovaSeq 6000 system (Illumina Inc., San Diego, CA, USA) in accordance with the manufacturer’s guidelines. The raw data were analyzed through the Sophia DDM data analysis platform. Variants were annotated using Sophia’s MOKA (Version 5.10.50.1) software. Alignment and variant discovery were performed using the Pepper basic algorithm based on the hg19 human genome reference.

The Genome Aggregation Database (gnomAD) (https://gnomad.broadinstitute.org, accessed on 1 August 2025), 1000 Genomes Project (http://www.1000genomes.org/, accessed on 1 August 2025), and Exome Aggregation Consortium (ExAC) (http://exac.broadinstitute.org/, accessed on 1 August 2025) were used to exclude variants with a minor allele frequency greater than 1%. The genes linked to the patient’s phenotype, particularly those involved in microcephaly, were prioritized for analysis. Variations with substantial impacts on the protein, including nonsense, frameshift, and canonical splice site variations, were initially assessed. The databases ClinVar (https://www.ncbi.nlm.nih.gov/clinvar, accessed on 1 August 2025), LOVD (https://www.lovd.nl, accessed on 1 August 2025), HGMD (http://www.hgmd.cf.ac.uk, accessed on 1 August 2025),and relevant publications were searched for potential variants. Following the guidelines established by the American College of Medical Genetics and Genomics (ACMG), the variants were finally classified according to the pathogenicities [15]. Segregation analysis of all variants within the families was performed using Sanger sequencing.

### 2.2. cDNA Preparation

First, 200 ng of total RNA was converted into cDNA by using the standard protocols of the IPSOGEN RT kit. Prepared cDNA samples were stored at −20 °C until the polymerase chain reaction (PCR) was conducted.

### 2.3. Primer Design and PCR

Region-specific primers were designed with PRIMER©—Primer Designer v.2.0 (Scientific & Educational Software programme) software. PCRs were carried out on these specific primers for each sample. RNA analysis was performed using RT-PCR, and the splicing effects of the intronic variants were evaluated by next-generation sequencing (MiSeq platform, Illumina, San Diego, CA, USA). Genomic DNA was prepared for next-generation sequencing using the Nextera XT sample preparation kit (Illumina Inc.). PCR products were purified with the NucleoFast^®^ 96 PCR kit (MACHEREY-NAGEL GmbH, Düren, Germany), quantified using the ND-1000 micro-volume spectrophotometer (Thermo Fisher Scientific Inc., Waltham, MA, USA), and normalized to 0.2 ng/µL, as required for library preparation. Libraries were sequenced on the MiSeq platform with a 300v2 cartridge, generating 2 × 150 bp paired-end reads (Illumina Inc.). The primer sequences and PCR conditions, including product size and annealing temperature, are provided in Supplementary Table S1.

Raw reads were aligned to the human reference genome (hg38) using BWA-MEM v0.7.17 [16]. Sorting, duplicate marking, and base recalibration were performed with GATK v4 [17]. Variant calling was conducted using both the UnifiedGenotyper and HaplotypeCaller algorithms from GATK. Aligned sequence data were visualized with the Integrative Genomics Viewer (IGV, Broad Institute, Cambridge, MA, USA).

## 3. Results

### 3.1. Clinical Features

**Figure 1:**
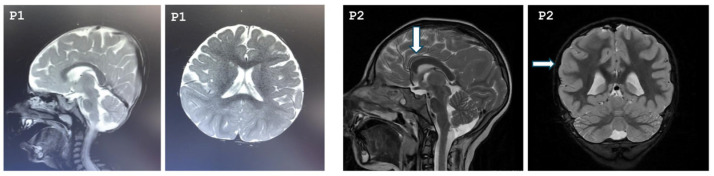
Cranial MRI images of Patient 1 and Patient 2. P1: T2-weighted sagittal and axial cranial MRI of Patient 1 demonstrating bilateral diffuse cerebral atrophy. P2: The arrows indicate a hypoplastic corpus callosum and pachygyria, respectively, in Patient 2.

Our study included seven patients diagnosed with MCPH3 from six different families. Three patients were females, and four were males. All patients were born to consanguineous parents who were first-degree cousins. The mean age of the patients was 7 years 3 months. Two of the patients were born late preterm, and all patients had birth weight and length appropriate for their gestational age. Compared to their motor skills, their speech development was more delayed. Two of the patients exhibited neuromotor developmental delay, and speech delay was noted in six patients. Five of the patients had learning difficulties and were receiving special education; evaluation was inconclusive for one patient (Patient 1) due to their young age. One patient had a history of seizures. On physical examination, all patients were found to have severe microcephaly, with a mean head circumference of −6.1 SDs (range: −7.9 to −3.7 SDs). Three of the patients (Patients 1, 3, 5, 7) exhibited postnatal short stature, while two patients had growth deficiency in weight (Patients 1, 6). On dysmorphic examination, the most prominent features observed in the patients were brachycephaly, hypertelorism, epicanthus, high-arched eyebrows, high nasal bridge, and micrognathia. Other dysmorphic findings were not distinctive and appeared to be patient-specific, such as sloping forehead, synophrys, upslanting palpebral fissures, ptosis, anteverted nares, low columella, long philtrum, and thick vermilion border. Conical teeth were observed in Patients 4 and 5. Additionally, the exfoliation of primary teeth had not yet occurred in Patient 5. Cutaneous pigmentary abnormalities of varying sizes in different body regions were observed in six of the seven patients. Cranial magnetic resonance imaging (MRI) revealed diffuse parenchymal volume loss in both cerebral hemispheres in Patient 1. In Patient 2, the frontal lobes appeared smaller than normal in both hemispheres, with associated findings of hypoplastic corpus callosum, pachygyria, and absence of the pineal gland. In Patient 6, an arachnoid cyst measuring 14 mm was detected in the retrocerebellar region. MRI findings were normal in the remaining patients. The cranial MRIs of Patients 1 and 2 are shown in Figure 1.

### 3.2. Common Findings

**Figure 2:**
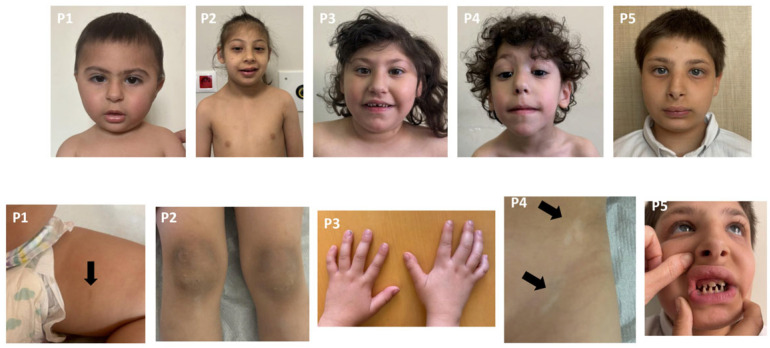
Dysmorphic facial features and various systemic involvements of the patients. All patients exhibited severe microcephaly. P1: High-arched eyebrows, synophrys, high nasal bridge. Hyperpigmented macule on the inner aspect of the left thigh. P2: High-arched eyebrows and high nasal bridge. Hyperkeratosis over the knees. P3: Short neck, high-arched eyebrows, upslanting palpebral fissures, hypertelorism, epicanthus, and high nasal bridge. Clinodactyly bilaterally. P4: High-arched eyebrows, hypertelorism, epicanthus, ptosis, high nasal bridge, and long philtrum. Hypopigmented macules, left lower abdomen. P5: Hypertelorism, epicanthus, high nasal bridge, anteverted nares, low columella, thick vermilion border, conical teeth, and micrognathia.

Almost all patients had severe microcephaly and mild to moderate intellectual disability. Chromosome and microarray analyses were normal. Metabolic investigations, including plasma acylcarnitine profiling, urinary organic acid analysis, plasma amino acid levels, and biochemical tests of liver and kidney function, were all within normal limits in the seven patients. All patients underwent normal abdominal ultrasonography, echocardiogram, and ophthalmological examination. The clinical, molecular, and radiological features of the patients are summarized in Table 1, and their photographs are shown in Figure 2.

### 3.3. CDK5RAP2 Variants

**Figure 3:**
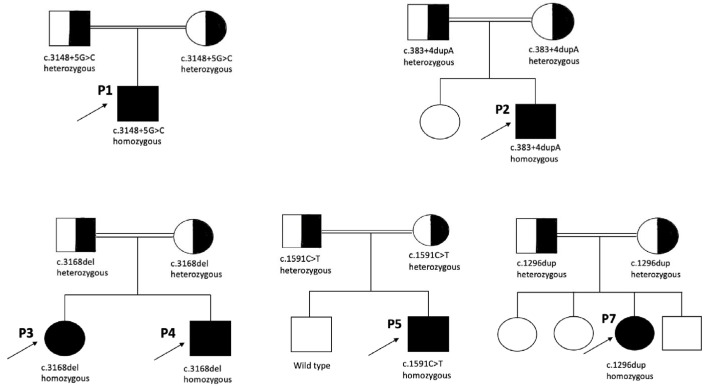
Pedigrees of the patients with *CDK5RAP2* variants.

We conducted WES and detected five different homozygous variants in the *CDK5RAP2* gene among the seven patients. Variant classification was carried out in accordance with ACMG recommendations. Two of the variants were intronic, while the others were frameshift and nonsense variants. The homozygous intronic variant c.3148+5G>C in Patient 1 has not been reported in population databases or previously associated with MCPH3 in the literature; however, it has been submitted to the ClinVar database as an uncertain significance (VUS) (VCV002000641.3). This variant fulfilled the PS3 and PM2 criteria based on the ACMG classification. In Patient 2, molecular analysis revealed an intronic variant, c.383+4dupA. The homozygous variant has not been previously reported in the literature. According to the ACMG criteria, the variant met PS3 and PM2. Both intronic variants were classified as likely pathogenic according to ACMG criteria. A homozygous frameshift variant, c.3168del (p.Asp1057Metfs*17), was detected in both siblings (Patient 3 and Patient 4). The novel variant has been considered pathogenic according to the ACMG criteria (PVS1, PP1, PM2). A homozygous nonsense variant, c.1591C>T (p.Gln531), was identified in Patient 5 and Patient 6. This novel variant has not been previously reported in the literature. Based on the ACMG guidelines, it fulfilled the PVS1 and PM2 criteria and was classified as likely pathogenic. A novel homozygous frameshift variant, c.1296dup (p.Asp433Argfs*6), was detected in Patient 7. Based on the ACMG guidelines, it fulfilled the PVS1 and PM2 criteria and was interpreted as likely pathogenic. Segregation analysis revealed that all variants were found to be heterozygous in the parents. Figure 3 depicts the pedigrees of patients carrying *CDK5RAP2* variants. The molecular characteristics of the patients are summarized in Table 1.

### 3.4. cDNA Analysis

cDNA analysis was performed on peripheral blood samples from Patient 1 and Patient 2. In Patient 1, the c.3148+5G>C variant was found to affect splicing, leading to the insertion of a 17-base pair (bp) intronic sequence from intron 23 (c.3148+1_3148+17) into the mRNA. The resulting aberrant mRNA introduces a premature stop codon at codon 1079. Therefore, this variant is expected to result in a loss-of-function effect.

In Patient 2, it was revealed that the c.383+4dupA variant affects normal splicing, resulting in the insertion of a 50 bp sequence from intron 5 (c.383+1_383+50) into the mRNA transcript. This aberrant splicing event introduces a premature stop codon at codon 129, leading to early termination of translation. Therefore, the c.383+4dup variant is predicted to have a loss-of-function effect on the gene product.

**Figure 4:**
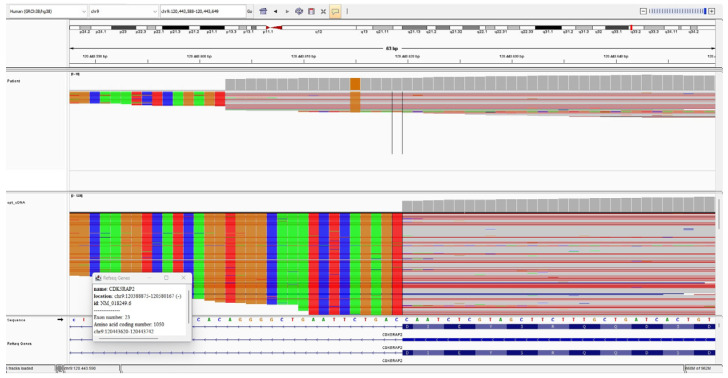
The IGV cDNA visualization of the c.3148+5G>C variant. The bold arrow indicates the c.3148+5G>C alteration. The thin arrow points to the 17-bp sequence inserted at the intron 23 start site.

**Figure 5:**
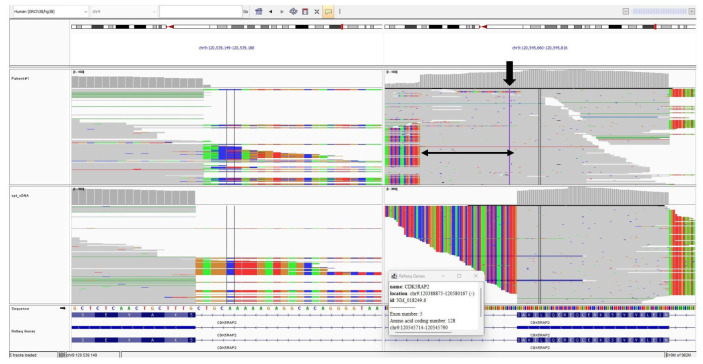
The IGV cDNA visualization of the c.383+4dup variant. The bold arrow indicates the c.383+4dup alteration. The thin arrow indicates the 50-bp sequence inserted from intron 5.

Both intronic variants fulfilled the PS3 criterion, as in vitro functional studies demonstrated their splicing defects. Figure 4 and Figure 5 show the IGV cDNA visualizations of the c.3148+5G>C and c.383+4dupA variants, respectively.

## 4. Discussion

In this study, we present seven patients from six unrelated families with biallelic *CDK5RAP2* variants, including four novel mutations, associated with MCPH3. MCPH3 is a very rare cause of primary microcephaly, with only a limited number of patients reported from Europe and Turkey. Consistent with previous reports, our patients exhibited severe congenital microcephaly, intellectual disability of variable degree, and speech delay.

*CDK5RAP2* (cyclin-dependent kinase 5 regulatory subunit-associated protein 2, MIM #608201) is a gene with 38 exons that encodes a protein of 1,893 amino acids [18]. *CDK5RAP2* controls CDK5 activity, which is necessary for centrosome cohesion, centriole duplication, and the anchoring of the centrosome to the spindle pole [19,20]. Biallelic pathogenic variants in this gene impair key processes, including centrosome formation and chromosome segregation, which in turn compromise neurogenesis [21]. Furthermore, it plays a crucial role in the cellular response to DNA damage [22]. *CDK5RAP2* is extensively expressed in the ventricular and subventricular regions during human neurogenesis, specifically at gestational weeks 12 and 18, and continues to be detected in the superficial neocortical layers through to full term [23].

The vast majority of patients with MCPH3 do not exhibit distinctive dysmorphic facial features. In a subset of patients, the most notable findings include a sloping forehead, hypertelorism, epicanthus, and a high nasal bridge [10,11,13,24]. In our patients, hypertelorism, epicanthus, and a high nasal bridge were the most prominent facial features, with the additional observation of high-arched eyebrows, a finding not previously reported in the literature. Nevertheless, as noted above, distinguishing patients based on phenotype alone is rarely possible. In most patients with MCPH3, neuromotor development, including head control, independent sitting, and walking, is appropriate for age; however, mild delays have been observed in some individuals [25,26]. Mild delays in neuromotor development were present in two of our patients (P1 and P3). In nearly all patients with MCPH3, mild to moderate intellectual disability is present, while severe impairment is observed in only a small number of cases [10,26,27]. In our cohort, one patient could not be reliably assessed due to young age, while another 4-year-old patient (P4) had no intellectual disability and had never received special education; however, no formal testing had been performed. In contrast, his older sister, carrying the same mutation, had intellectual disability and was receiving special education. Cranial MRI findings in MCPH3 patients include reduced brain volume, gyral simplification, agenesis, hypoplasia, or dysgenesis of the corpus callosum, and enlargement of the cisterna magna. In the study by Erdogan et al. (2025) [10], previously unreported findings such as an arachnoid cyst and colpocephaly were documented. Nasser et al. (2020) [26] identified inter-hypothalamic adhesion (IHA) in 5 of 7 patients; however, none of their patients exhibited signs suggestive of hypothalamic dysfunction, such as micropenis, diabetes insipidus, or hypertension. IHA has not been reported in any MCPH3 patient prior to these observations. Nevertheless, when particularly thin or small, IHA may not always be detectable on routine brain MRI. Therefore, high-resolution imaging protocols with thin slices should be employed. All of our patients underwent cranial MRI, with pathological findings detected in only three patients. In Patient 1, diffuse parenchymal volume loss was observed in both cerebral hemispheres, whereas Patient 2 presented with reduced frontal lobe volumes, hypoplastic corpus callosum, pachygyria, and absence of the pineal gland. To the best of our knowledge, pachygyria and absence of the pineal gland have not been previously reported in other MCPH3 patients. In Patient 6, a retrocerebellar cyst measuring 14 mm was detected, similar to the finding reported by Erdogan et al. (2025) [10]. These findings all suggest that cranial involvement in MCPH3 is likely heterogeneous. To the best of our knowledge, seizures have been reported in 26% of patients with MCPH3 in the literature, including generalized tonic–clonic seizures and infantile spasms [10,11,12,13,14,18,23,24,25,27,28,29,30,31,32]. Consistent with the literature, one of our patients had generalized tonic–clonic seizures that began at the age of four. Only a small number of MCPH3 patients in the literature have undergone cranial MRI. Therefore, we could not obtain sufficient information to determine whether there is an association between seizures and cranial involvement. However, in the literature, patients with seizures who underwent cranial MRI were found to have non-specific findings—such as enhancement in the white matter, dilated ventricular system, thinning of the corpus callosum, and cerebral volume loss—which were also observed in patients without seizures [14]. Ataxia has been reported in only a few patients to date in the literature [13,26]. In the study by Jouan et al. (2016) [33], ataxia was observed in three patients with biallelic *CDK5RAP2* mutations who presented with mild learning disability and agenesis of the corpus callosum, but without microcephaly. In our patient cohort, ataxia was identified in two individuals. As the mutations in our patients were novel, comparison with previously reported cases in the literature was not possible.

In MCPH3, microcephaly is typically severe, whereas intellectual disability is generally mild to moderate, and neuromotor delay is uncommon. Most patients adapt well to daily life with supportive educational programs. Although rare cases with severe intellectual disability, spasticity, muscle atrophy, and flexion contractures have been reported, their central nervous system involvement appears similar to that of milder cases [10,14,26]. While WES has been performed in all reported patients, microarray analysis has not, raising the possibility of unrecognized copy number variations or reflecting a more severe phenotype of the disease. Notably, no severe cases were observed in our cohort.

So far, the Human Gene Mutation Database (HGMD Professional 2025.2, April 2025), including the study by Erdogan et al. (2025) [10], has documented nearly 40 disease-causing mutations in the *CDK5RAP2* gene associated with primary microcephaly in affected individuals. Among these mutations, frameshift variants represent 39%, nonsense variants 31%, missense variants 6%, and intronic variants 6%, with the majority of the latter located at canonical splice acceptor/donor sites. Among the mutations we identified in our patients, two were frameshifts, one was a nonsense, and the remaining two were intronic. Since the families of Patients 5 and 6 harbored the same nonsense mutation (c.1591C>T p.Gln531), they were questioned about possible consanguinity; although they reported being from the same hometown, they stated that they were not related. Considering the rarity of *CDK5RAP2* mutations and their shared place of origin, a distant relationship through previous generations is suspected. No hotspot region of the *CDK5RAP2* gene has been identified in association with MCPH3 in the literature. A detailed review of the literature revealed that the mutations are distributed homogeneously throughout the whole gene; however, exons 7, 12, and 30 harbor a higher number of mutations compared to other exons. In our cohort, the c.1296dup variant identified in Patient 7 was also located in exon 12. The majority of mutations identified in *CDK5RAP2* result in loss of function. In our study, all identified variants, including intronic variants, were predicted to undergo nonsense-mediated decay (NMD), as none were located in the last exon or within the last 50 bp of the penultimate exon.

Intronic variants can exert a wide range of functional consequences on gene expression and protein function. Variants located near canonical splice donor or acceptor sites may lead to aberrant splicing events such as exon skipping, intron retention, or the creation of cryptic splice sites. These alterations can generate frameshifts or premature termination codons, ultimately resulting in truncated or non-functional proteins through NMD. In addition, intronic changes may disrupt splicing enhancers or silencers, thereby altering the balance of transcript isoforms and affecting protein structure or abundance. Collectively, these mechanisms highlight the importance of evaluating intronic variants not only by in silico prediction tools but also by functional RNA analyses, in order to establish their true pathogenic impact. We performed RNA analysis to investigate the effects of the c.3148+5G>C and c.383+4dupA intronic variants on splicing. RNA analysis demonstrated that both identified intronic variants had a direct impact on normal splicing. In Patient 1, the c.3148+5G>C variant resulted in the insertion of a 17-bp intronic sequence into the mRNA, which introduced a premature stop codon at codon 1079. In Patient 2, the c.383+4dupA variant caused the inclusion of a 50-bp sequence from intron 5, leading to a premature stop codon at codon 129. We found that both variants disrupted splicing, leading to the generation of a premature stop codon and resulting in early termination of the protein. Thus, we demonstrated that these variants result in a loss-of-function effect. These findings provide functional evidence that intronic mutations, although located outside the canonical splice sites, can critically disrupt splicing and lead to truncated, non-functional proteins, thereby contributing to the pathogenesis of MCPH3. Interestingly, in half of the six identified intronic mutations in the literature, the variants were located at the −1, −9, and −15 nucleotides upstream of exon 27 (c.4005-1G>A; c.4005-9A>G; c.4005-15A>G). Yigit et al. (2015) [31] functionally investigated the intronic variants c.4005-9A>G and c.4005-15A>G in *CDK5RAP2* and demonstrated that both led to alternative splicing, resulting in a frameshift and a premature stop codon. These results, together with our data, further highlight the pathogenic potential of intronic variants in *CDK5RAP2* and their role in the etiology of MCPH3. Various in silico prediction tools, such as SpliceAI, have been developed to estimate the effects of intronic variants on splicing. As these are algorithm-based, they do not provide definitive information but rather predictive results. Functional studies are essential for the interpretation of variants of uncertain significance, particularly intronic ones. Such analyses can clarify whether a variant disrupts splicing or alters gene expression, thereby distinguishing truly pathogenic variants from benign changes. Including functional validation is therefore critical for accurate genetic diagnosis and counseling. In our study, we advanced this further by clarifying the precise impact of the variants. Traditionally, genetic diagnostics have primarily focused on exonic mutations, as they are more straightforward to interpret. However, our findings highlight that intronic variants can also disrupt splicing regulatory elements and generate aberrant transcripts, thereby playing a crucial role in disease etiology. This underscores the importance of systematically evaluating non-coding regions in addition to exons in order to achieve accurate molecular diagnoses.

This study has several limitations. First, the number of patients included was relatively small. Additionally, although one of our patients exhibited typical clinical findings of the disease, intellectual disability was not observed; however, no standardized intelligence testing was performed to confirm this observation. The genotype–phenotype correlation could not be fully addressed, both because of the limited sample size and the presence of novel variants. Finally, we did not perform functional validation of the identified variants using cellular or animal models, which may have further clarified their pathogenic mechanisms.

## 5. Conclusions

The diagnosis and genetic counseling of patients with microcephaly, intellectual disability, and speech delay, who lack significant systemic involvement, are especially important for future generations. Our findings underscore the importance of including intronic regions in genetic testing panels for primary microcephaly. Intronic variants can critically disrupt splicing and lead to aberrant transcripts, highlighting that analysis limited to exonic regions may fail to capture clinically relevant pathogenic mutations. Furthermore, accurate identification of the underlying genetic disorder is essential for providing effective genetic counseling. Given the considerable risk of recurrence, preimplantation genetic testing should be offered to affected families.

## Figures and Tables

**Table 1:** Clinical and molecular findings of patients.

Patient	Family 1/Patient 1	Family 2/Patient 2	Family 3/Patient 3	Family 3/Patient 4	Family 4/Patient 5	Family 5/Patient 6	Family 6/Patient 7
**CDK5RAP2 homozygous variant (NM_018249.6)**	c.3148+5G>C	c.383+4dupA	c.3168del (p.Asp1057Metfs*17)	c.3168del (p.Asp1057Metfs*17)	c.1591C>T (p.Gln531*)	c.1591C>T (p.Gln531*)	c.1296dup (p.Asp433Argfs*6)
**Variant type**	Intronic	Intronic	Frameshift	Frameshift	Nonsense	Nonsense	Frameshift
**ACMG pathogenicity criteria**	PS3, PM2	PS3, PM2	PVS1,PM2,PP1	PVS1,PM2,PP1	PVS1,PM2	PVS1,PM2	PVS1,PM2
**Novel variant**	-	**+**	**+**	**+**	**+**	**+**	**+**
**Gender**	M	F	F	M	M	M	F
**Consanguineous marriage**	+	+	+	+	+	+	+
**Gestational age**	36 + 6	39 + 4	40 + 1	39	38	41 + 2	37 + 5
**Birth weight**	2200 g	2910 g	2650 g	2800 g	3000 g	3650 g	3200 g
**Birth length**	42 cm	49 cm	48 cm	49 cm	?	50 cm	?
**Birth head circumference**	30 cm	28 cm	29 cm	33 cm	?	32.5 cm	?
**Age**	15 months	6 years 3 months	6 years 8 months	4 years	12 years	7.5 years	14 years
**Head circumference SDS**	−5.5	−6.7	−5.7	−3.7	−7.9	−6.4	−7
**Height SDS**	−2	−1.2	−2	−1.5	−3.3	−1.6	−3.4
**Weight SDS**	−2.5	−1.2	1	−1.1	−1.4	−3.5	2
**Short stature**	+	-	+	-	+	-	+
**Facial gestalt**	Brachycephaly, high-arched eyebrows, synophrys, high nasal bridge	High-arched eyebrows and high nasal bridge	Short neck, high-arched eyebrows, upslanting palpebral fissures, hypertelorism, epicanthus, and high nasal bridge	High-arched eyebrows, hypertelorism, epicanthus, ptosis, high nasal bridge, and long philtrum	Brachycephaly, hypertelorism, epicanthus, high nasal bridge, anteverted nares, low columella, thick vermilion border, and micrognathia	Sloping forehead, mild micrognathia	-
**Sitting age**	12 months	7 months	9 months	7 months	?	7-8 months	7 months
**Independent walking**	-	12 months	18 months	10 months	12 months	12 months	11 months
**First words**	-	2 years	4 years	12 months	3 years	2.5 years	2 years
**Intellectual disability**	?	+	+	-	+	+	+
**Speech delay**	+	+	+	-	+	+	+
**Seizures**	-	+	-	-	-	-	-
**Ataxia**	?	-	+	-	+	-	-
**Hearing loss**	-	+	+	-	-	-	+
**Skin involvement**	Hyper/hypopigmentation lesions	Hyper/hypopigmentation lesions	Hyperpigmentation lesions	Hyper/hypopigmentation lesions	Hyper/hypopigmentation lesions	-	Hyperpigmentation lesions
**Tooth involvement**	-	-	-	Conical teeth	Conical teeth, microdontia, and persistence of primary teeth	-	-
**Cranial MRI**	Diffuse parenchymal volume loss in both cerebral hemispheres	The frontal lobes appear smaller than normal in both cerebral hemispheres, hypoplastic corpus callosum, pachygyria, absence of pineal gland	Normal	Normal	Normal	Retrocerebellar arachnoid cyst	Normal
**Attention deficit and hyperactivity**	-	-	+	+	-	-	-
**Other anomalies**		Asymmetrically positioned nipples and hyperkeratosis over the knees	Clinodactyly and increased deep tendon reflexes	Brachydactyly	Hypermobile fingers		

## Data Availability

The original contributions presented in the study are included in the article, further inquiries can be directed to the corresponding author.

## Supplementary Materials

The following supporting information can be downloaded at: https://www.mdpi.com/article/10.3390/genes16101120/s1, Table S1: Primer sequences designed for the patient; Table S2: PCR reaction content; Table S3: PCR conditions.

## Abbreviations

ACMG	American College of Medical Genetics and Genomics
HPO	Human Phenotype Ontology
IGV	Integrative Genomics Viewer
IHA	identified adhesion
MCPH	Microcephaly primary hereditary
MCPH3	Primary Autosomal Recessive Microcephaly 3 (MCPH3)
MRI	Cranial magnetic resonance imaging
PM	Primary microcephaly
WES	Whole-exome sequencing
