# Supplementary material for: Expanding the Clinical and Molecular Spectrum of Primary Autosomal Recessive Microcephaly: Novel CDK5RAP2 Gene Variants and Functional Insights on the Intronic Variants
Source: Genes (Basel). 2025 Sep 23;16(10):1120. doi: 10.3390/genes16101120 (PMC12564466; doi:10.3390/genes16101120)
Supplement: Supplementary file 1 [file genes-16-01120-s001.zip › genes-3870153-supplementary.pdf]

Table S1. Primer sequences designed for the patient

Primer	Orientation	Sequence (5'->3')
**CDK5RAP2_cDNA_F**	Forward	CCCTGGTATTACCAACAGAGAGGCTAAG
**CDK5RAP2_cDNA_R**	Reverse	CTAATGAATCGTATCTGGGAGGTGAGAG

Table S2. PCR reaction content

Content	For each reaction (µl)
dH_20	15
5x Buffer Solution (Thermo Inc.)	5
dNTP mix, each 10mM	0,5
Forward Primer (5 µM)	1,5
Reverse Primer (5 µM)	1,5
PhireII HS DNA Polimerase (Thermo Inc.)	0,5
cDNA sample	1
**Total**	**25**

Table S3. PCR conditions

<table>
<tr><th>Temperature (^{0}C)</th><th>Duration (mm:ss)</th><th>Cycles</th></tr>
<tr><td>95</td><td>1:00</td><td>1</td></tr>
<tr><td>95</td><td>00:10</td><td rowspan="3">45</td></tr>
<tr><td>60</td><td>00:10</td></tr>
<tr><td>72</td><td>00:20</td></tr>
<tr><td>72</td><td>1:00</td><td>1</td></tr>
<tr><td>4</td><td>∞</td><td>1</td></tr>
</table>
